# Progress in Surgery

**Published:** 1901-11-16

**Authors:** 


### Progress in Surgery.

(*Continued from page* 100.)

**The Diagnosis of Renal Disease.**—Kümmell[7] (Hamburg) has published an account of his further observations and experiences of the method of ascertaining the functional efficiency of the kidneys by finding the freezing-point of the blood and of the urine. He has determined the freezing-point of the blood a hundred times, and also many times that of the urine with the help of catheterisation of the ureters. The results have been controlled by examinations of the kidneys either at operations or at autopsies. Human blood normally freezes at ·56°C. below zero. Variations between ·55° and ·57° may be regarded as physiological. A lowering to ·58° or more below zero indicates renal insufficiency. No operative interference on the kidneys should be undertaken unless the freezing point of the blood is not lower than about ·56° below zero. The determination of these freezing points, in conjunction with the estimation of urea, and of the sugar found after the administration of phloridzin, in the urine obtained from each ureter, settles the question of renal sufficiency with great certainty, and shows clearly when operations may be performed on the kidneys with safety. If the blood freezing-point be taken after removal of a diseased kidney, and found constant, it shows that no nitrogenous bodies are being retained in the tissues as a result of the operation. The lower the freezing point of the urine drawn off from a ureter and the less the quantity of urea found in it, the more has the kidney tissue been destroyed by the disease. Kümmell relates cases which verify this statement. Through the application of the above methods Kümmell has avoided the anuria which is so much feared after excision of the kidney. But such an anuria may follow operations on one kidney, although the second kidney was previously sound. E. Fränkel has shown that there may result from the operation, especially from the effects of the anæsthetic, a marked degeneration of the renal epithelium. This can only be demonstrated by the microscope, no changes being detectable by the naked eye. These anatomical changes explain the so-called "reflex anuria" which sometimes follows operations even on other parts of the body than the kidney. Kümmell's observations have confirmed those of Von Korányi, that large tumours of the kidney, including pyoneprosis, etc., or of other abdominal organs, may cause a depression of the blood-freezing point, even though there is renal sufficiency. In such cases the phloridzin test, and the determination of the freezing point of the urine, and of the amount of urea excreted, will abolish doubt. M. P. Bazy[8] draws attention to certain signs which help in the diagnosis of suppurative lesions of the kidney. It is held to be important to study these simple and practical methods of diagnosis, because cystoscopy and catheterisation of the ureters is often impracticable. He deals with cases in which there is frequent micturition, terminal pain, and turbid urine. If pain radiates towards the bladder, and if pressure on the anterior abdominal wall, about an inch to the right or left of the median line, causes a desire to micturate, it indicates pyelitis. This sign, called the pyelo-vesical reflex, is rarely obtained. The uretero-vesical reflex is more frequently found. It is difficult to obtain in the male, however. It consists in the discovery of tenderness of the ureteral opening to pressure applied through the bladder, vagina, or rectum. Such tenderness also shows kidney disease on the same side. If cystitis only is present then only the bladder-neck will be painful on pressure. If purulent urine is added to Fehling's solution until a pale blue or green colour is produced, on shaking the mixture small gas bubbles will form if the pus is of renal origin, and on boiling the coagulum will in consequence rise to the top. If the pus is of bladder origin there will be no gas bubbles and the coagulum will fall to the bottom.

**Anatomical Associations of the Kidneys.**—Edmund Owen[9] has published an interesting paper on this subject. He points out that the renal nerves are derived from the sympathetic plexus in front of the aorta, which plexus receives branches from the splanchnics, pneumo-gastrics, and from the spinal nerves. On the other hand the plexus gives branches to all the viscera, and if disturbance should arise in any one viscus, *e.g.* in the kidney from stone, it is therefore not surprising to find that the brain does not always locate the source of mischief. Thus errors in diagnosis are apt to arise. As an illustration the writer quotes the case of a man who five years previously had left renal colic, and had had other similar attacks since. But for the last two years all the pain had been on the right side, and had been very severe. The patient was quite convinced that small calculi he had passed came from the right side. The right kidney was explored and found to be healthy. Six weeks later a branching calculus was removed from the left side. After a second exploration about six months later, finally, after a sinus had persisted another twelve months, the kidney was removed. The writer then refers to the value of a score of leeches, or cupping of the loins, in assisting diseased kidneys to do their work. This is explained by the vascular associations of the kidneys. Robert Harrison and Sir William Turner have shown that there is a subperitoneal arterial plexus which anastomoses freely with the visceral arteries. The phrenic, lower intercostal, and upper lumbar arteries are the chief vessels in this anastomosis. In illustration of the relations of the kidney to the colon, a case is mentioned of a man who had back pains, and pain passing down into the left thigh and testis, and marked tenderness in the left renal region, and a history of hæmaturia. The kidney was found to be healthy on exploration. Later, at a post-mortem examination, an epithelioma was found growing into the descending colon on a level with the kidney and secondary growths in the liver. Another instance mentioned is that of a man with carcinoma of the colon, who had been operated upon by another surgeon for movable kidney owing to an error in diagnosis. Also the writer quotes the case recorded by C. A. Morton, in which a movable abdominal swelling was thought to be the kidney, but exploration showed that organ to be normal and that the swelling was due to malignant disease of the cæcum. The latter was successfully excised. In conclusion, the author strongly advocates giving nature a chance to repair a traumatic rupture of the kidney, even if exploration shows that the kidney has been completely torn across.

**The Surgical Treatment of Renal Tension.**—Reginald Harrison[10] recently discussed this subject. If in scarlatinal nephritis, after a few weeks the signs of nephritis—such as albuminuria and casts—do not disappear then surgical interference is desirable. It is also applicable to the cases in which the renal attack is of a malignant type, in which suppression of urine occurs, and death rapidly follows from uræmia with coma and convulsions. The writer's method of operating is to incise the capsule along the convex border, and to make punctures anywhere where engorgement seems greatest, avoiding, however, the renal pelvis. Blood often spurts out in jets through the punctures. A drainage tube must be carefully inserted so as to lie in contact with the organ, and must be retained for some days, or even weeks. The relief of tension in one kidney seems to aid the other, and the urine has on several occasions been noticed to become normal in quantity and quality. In a similar way in traumatic injury of the kidney the renewal of the pressure of extravasated blood may restore renal secretion in threatened or actual anuria.

[7] Beilage z. Centralb. f. Chir., 1901, No. 29. [8] *Vide* Epitome Brit. Med. Journ., May 18, and Centralb. f. Chir., July 27, 1901. [9] Brit. Med. Journ., June 8, 1901, p. 1385. [10] Lancet, Aug. 3, 1901, p. 330.

